# Four SARS-CoV-2 Genome Sequences from Late April in Stockholm, Sweden, Reveal a Rare Mutation in the Spike Protein

**DOI:** 10.1128/MRA.00934-20

**Published:** 2020-08-27

**Authors:** Tatiany Aparecida Teixeira Soratto, Hamid Darban, Annelie Bjerkner, Maarten Coorens, Jan Albert, Tobias Allander, Björn Andersson

**Affiliations:** a Department of Cell and Molecular Biology, Karolinska Institutet, Stockholm, Sweden; b Department of Microbiology, Immunology, and Parasitology, Federal University of Santa Catarina, Florianopólis, Santa Catarina, Brazil; c Department of Microbiology, Tumor and Cell Biology, Karolinska Institutet, Stockholm, Sweden; d Department of Clinical Microbiology, Karolinska University Hospital, Stockholm, Sweden; DOE Joint Genome Institute

## Abstract

Here, we report four coding-complete severe acute respiratory syndrome coronavirus 2 (SARS-CoV-2) genome sequences from Stockholm, Sweden, sampled in late April 2020. A rare variant at bp 23463 of the SARS-CoV-2 genome was found, which corresponds to the S1 subunit of the spike protein, changing an arginine (R) residue to histidine (H).

## ANNOUNCEMENT

A severe pneumonia disease caused by severe acute respiratory syndrome coronavirus 2 (SARS-CoV-2), a virus that belongs to the family *Coronaviridae* and the genus *Betacoronavirus*, emerged in Wuhan, China, in December 2019 and has rapidly spread around the world (1, 2). As this virus is new to humans, large research efforts are going into characterizing the virus, mapping its spread, and studying its biological and clinical features. We report here four SARS-CoV-2 genome sequences obtained from patients confirmed to have the disease. The sampling and tests were carried out on 26 April 2020 at the Karolinska University Hospital in Stockholm, Sweden. The study was approved by the local ethics committee at the Karolinska Institute, The Regional Ethical Review Board (reference numbers 02-212, 02-422, and 04-836/4).

Nasopharyngeal swab specimens were collected from 23 patients suspected to have coronavirus disease 2019 (COVID-19), the disease caused by SARS-CoV-2. In 17 of these, a reverse transcriptase PCR (RT-PCR) assay for SARS-CoV-2 (3) yielded a positive result, with cycle threshold (*C_T_*) values ranging from 11 to 35. Viral RNA was extracted, and cDNA was synthesized using the QIAseq FX single-cell RNA library kit (Qiagen). Illumina libraries with an average length of 350 bp were prepared using the ThruPLEX DNA-seq kit (Rubicon Genomics) and sequenced using Illumina MiSeq technology (2 × 300 bp). The genomes were assembled, and nucleotide variants were assigned for each genome using the Genome Detective virus tool, version 1.126 (4), using SARS-CoV-2 (GenBank accession number MN908947.3) as the reference.

SARS-CoV-2 reads were detected in nine of the samples, with variable coverage. Near-complete genomes were assembled from four samples, with a median length of 29,825 ± 7 bp, covering 99.7 to 99.8% of the reference genome and 100% of the coding region, with depths of coverage ranging from 776.8 to 1,718.2× and 38.00% GC content. An additional genome assembly covered 80.6% in 17 contigs with an average depth of coverage of 12.2× and 38.30% GC content.

**FIG 1:**
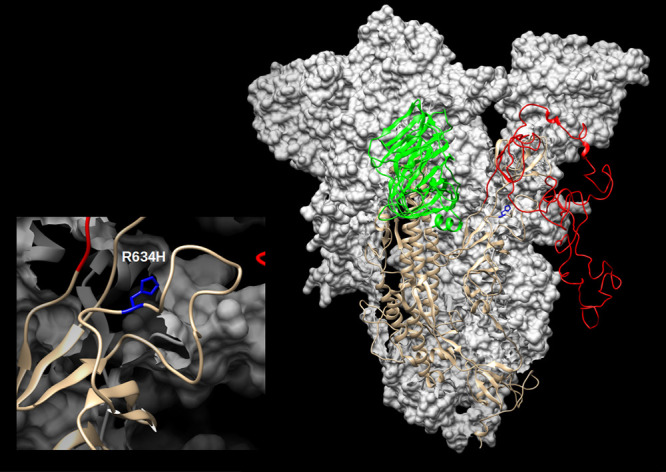
SARS-CoV-2 spike (S) modelled with SWISS-MODEL (9) using the 6ZGH structure as a template, drawn and colored in UCSF Chimera (10). The N-terminal domain (NTD) is colored green, and the receptor-binding domain/C-terminal domain (RBD/CTD) is red. The enlarged inset shows the location of the R634H mutation (blue).

The four coding-complete genomes each have 9 to 14 single nucleotide differences compared to the reference. All four genomes have mutations in noncoding position 241 C to T and have three mutations in coding regions, two C to T in positions 3037 and 14408 and one A to G in position 23403 (Table 1). The variant at bp 23463, found at the P17157_1020 genome, was not found in any other SARS-CoV-2 genome that was present in GISAID and GenBank at the time that this report was drafted (13 July 2020). The impact of this spike protein R364H variant (Fig. 1) was predicted by the DUET Web server (5) to have destabilizing effects. The variant is located at the surface of the S1 subunit and could possibly affect the attachment of the virion to the cell, even though it does not change the receptor-binding domain itself (Fig. 1).

**TABLE 1:** Mutations detected in the SARS-CoV-2 strains in this study

Nucleotide position	Ref. Base^*a*^	Mutant base	Mutation found in strain:					Gene	Type of mutation
			P17157_1021	P17157_1020	P17157_1016	P17157_1007	P17157_1018^*b*^		
219	G	T	T	G	G	G	G		Noncoding
241	C	T	T	T	T	T	T		Noncoding
1059	C	T	T	T	T	C	T	ORF1a	T265I
2659	G	T	G	G	G	T	G	ORF1a	K798N
2755	G	T	G	T	G	G	G	ORF1a	Synonymous
3037	C	T	T	T	T	T	T	ORF1a	Synonymous
3184	A	G	A	A	G	A	A	ORF1a	Synonymous
5147	C	T	C	C	C	T	C	ORF1a	R1628C
6285	C	T	C	C	C	T	C	ORF1a	T2007I
6352	G	T	G	T	G	G	G	ORF1a	K2029N
9193	A	G	A	A	A	G	A	ORF1a	Synonymous
11083	G	T	G	G	G	T	G	ORF1a	L3606F
11398	T	G	T	T	T	G	T	ORF1a	Synonymous
12915	C	T	T	C	C	C	C	ORF1a	T4217I
14408	C	T	T	T	T	T	T	ORF1ab	P4715L
21575	C	T	C	C	T	C	C	S	L5F
23202	C	T	T	C	C	C	C	S	T547I
23403	A	G	G	G	G	G	G	S	D614G
23463	G	A	G	A	G	G	G	S	R634H
24368	G	T	T	T	T	G	G	S	D936Y
25563	G	T	T	T	T	G	T	ORF3a	Q57H
27549	C	T	C	C	C	T	C	ORF7a	Synonymous
28881	G	A	G	G	G	A	G	N	R203K
28882	G	A	G	G	G	A	G	N	R203K
28883	G	C	G	G	G	A	G	N	G204R
28889	T	C	T	C	T	T	T	N	S206P
29287	A	T	T	A	A	A	A	N	K338N

a Ref., reference.

b Only accepted mutations found in the coding-complete genomes.

We compared the four coding-complete SARS-CoV-2 genomes from Stockholm with genomes detected globally using the three main methods available to establish relationships between different genetic variants of the virus, GISAID, Nextstrain, and PANGOLIN (Phylogenetic Assignment of Named Global Outbreak LINeages) (6, 7). According to all three tools, the Stockholm genomes belonged to two genetic groups, 20C/B.1/G and 20B/B.1.1/GR. These groups were reported by the Public Health Agency of Sweden as two of the three main genetic groups found in Sweden (8). Somewhat surprisingly, three of the coding-complete genomes described here are from group B.1/G, which was reported to have declined in prevalence by late April. This lineage has spread to more than 20 countries in Europe, the Americas, Asia, and Australia and corresponds to the Italian outbreak.

### Data availability.

These sequences have been deposited in the European Nucleotide Archive (ENA) under the study reference number PRJEB39632. The reads of the five SARS-CoV-2 strains were deposited in the ENA under the accession numbers ERR4387391, ERR4387389, ERR4387388, ERR4387386, and ERR4387385. The consensus sequences were also deposited under the accession numbers ERZ1478570, ERZ1478358, ERZ1478357, ERZ1478356, and ERZ1478355.
